# A comparison of reinforcement learning models of human spatial navigation

**DOI:** 10.1038/s41598-022-18245-1

**Published:** 2022-08-17

**Authors:** Qiliang He, Jancy Ling Liu, Lou Eschapasse, Elizabeth H. Beveridge, Thackery I. Brown

**Affiliations:** 1 School of Psychology, Georgia Institute of Technology, Atlanta, USA; 2 School of Economics, Georgia Institute of Technology, Atlanta, USA

**Keywords:** Human behaviour, Computational science

## Abstract

Reinforcement learning (RL) models have been influential in characterizing human learning and decision making, but few studies apply them to characterizing human spatial navigation and even fewer systematically compare RL models under different navigation requirements. Because RL can characterize one’s learning strategies quantitatively and in a continuous manner, and one’s consistency of using such strategies, it can provide a novel and important perspective for understanding the marked individual differences in human navigation and disentangle navigation strategies from navigation performance. One-hundred and fourteen participants completed wayfinding tasks in a virtual environment where different phases manipulated navigation requirements. We compared performance of five RL models (3 model-free, 1 model-based and 1 “hybrid”) at fitting navigation behaviors in different phases. Supporting implications from prior literature, the hybrid model provided the best fit regardless of navigation requirements, suggesting the majority of participants rely on a blend of model-free (route-following) and model-based (cognitive mapping) learning in such navigation scenarios. Furthermore, consistent with a key prediction, there was a correlation in the hybrid model between the weight on model-based learning (i.e., navigation strategy) and the navigator’s exploration vs. exploitation tendency (i.e., consistency of using such navigation strategy), which was modulated by navigation task requirements. Together, we not only show how computational findings from RL align with the spatial navigation literature, but also reveal how the relationship between navigation strategy and a person’s consistency using such strategies changes as navigation requirements change.

## Introduction

Reinforcement learning (RL) has made tremendous progress in the past two decades in computer science, psychology and neuroscience^1–5^. RL describes a learning mechanism in which behaviors are shaped through approaching rewards and avoiding punishments, which has a long history dating back to the nineteenth century^6^. In psychology, RL studies show that RL can be hierarchically structured^7^ and it interacts with other cognitive functions such as working memory in decision-making^8–10^. In neuroscience, RL studies have sought to reveal the neural substrates representing RL parameters (e.g., prediction error in the RL model), which include the striatum^11,12^, the ventromedial prefrontal cortex^13^, hippocampus^14^ and the firing behavior of dopamine neurons^15^. Most of the paradigms used in human RL studies focus on decision-making in 2D environments, so it remains understudied whether these findings can be generalized to scenarios important for survival like spatial navigation, which takes place in 3D environments. Moreover, modeling human spatial navigation behaviors via RL models could provide additional insight into the underlying cognitive mechanisms for a given navigational route compared to traditional methods (e.g., computing a route’s length) by using a person’s own navigational history to quantify that individual’s navigation strategies and their consistency of using such strategies (discussed more below). The current study aims to address these gaps and opportunities in the literature.

To maximize rewards while minimizing effort/cost, we learn the associations between behaviors and rewards mainly through two types of RL: model-free—which fosters rigid repetitions of previously rewarded actions, or model-based—which fosters a mental model of the environment or task structure to flexibly select goal-directed actions^11,12,16,17^. In spatial navigation, model-free learning corresponds to response learning, or merely learning the landmark-action associations (e.g., turn right at the second traffic light) without learning the overall layout of the environment. Model-based learning, on the other hand, corresponds to place learning or learning the configuration of the environment^12,18,19^. From the RL literature, there is evidence showing that model-free and model-based learning operate and compete in parallel^9,11,20,21^. From the human spatial cognition literature, there is a plethora of evidence showing there are substantial individual differences in human spatial navigation, including wayfinding performance^19,22–28^ and navigation strategy^29–34^. Such individual differences are found to be correlated with structural and functional differences of the brain^34–39^, working memory capacity^23,27,40^, gender^29,41^ and task instructions^31,33^. Taking the findings from RL and spatial cognition together, it is reasonable to hypothesize that whereas the navigation behaviors of some people are best fit by either the model-free or the model-based learning, the majority of them should be best fit by a hybrid model, which is a combination or more-continuous balance of model-free and model-based learning.

Surprisingly, there were very few studies lending support to this idea that such a hybrid RL model provides the best fit to characterize human spatial navigation. Using a spatial navigation task where the layout of the environment changed continuously, Simon and Daw^12^ found that the model-based learning fit the data better than the model-free, but whether a hybrid model provides a better fit than the model-based model remains unknown. Using several spatial navigation tasks, Anggraini et al.^18^ compared the neural substrates which tracked model-free, model-based and hybrid parameters, but which model provides the best fit to the navigation data themselves remains unknown. We believe that comparing the model-free, model-based and the hybrid models is crucial for efforts to investigate how the results from computational modeling, such as RL, fit the well-established individual differences and strategy-preference findings in the spatial cognition literature.

If the results from RL are in line with the findings from the literature, then we can harness the parameters generated from RL models to better understand human spatial navigation with much more confidence. As noted above, one unique contribution of applying RL to understanding human spatial navigation is that RL models can reveal one’s navigation strategy quantitatively and as a function of one’s prior experiences. In recent literature, we and others have used the dual-solution task^30^ to quantify individuals’ navigation strategies towards following a familiar route vs. taking a shortcut. In this approach, participants first follow a pre-defined route to a destination several times, and then they can navigate to the destination freely. Using this task, individual navigation strategy is quantified via the solution index, which is equal to the number of trials in which a novel shortcut is taken divided by the number of successful trials in which either a shortcut or the learned route is taken. Studies using this task show marked individual differences in navigation tendency, such that some individuals primarily use shortcuts, some primarily use familiar routes, and many fall between these extremes^30,31,34^. However, classifying the navigation strategy of an episode into either familiar route following or shortcut taking in this manner may simplify many complex navigation behaviors observed in our daily life. It is often possible that one first follows the first few sections of the learned route and then takes a shortcut from there; similarly, the cognitive demands of a “shortcut” may depend heavily on how much of the route sequence has been previously traversed (i.e., to what degree is the route a novel construct). The weight parameter of a RL hybrid model can reveal a navigator’s reliance on cognitive map vs. route-following on a trial-by-trial basis, which provides a finer grained characterization of one’s navigation strategy in an objective and continuous manner, based on their prior navigation history. Furthermore, RL models can also estimate the consistency of using a navigation strategy across different navigation episodes. These two parameters from RL provide important insight into how one adapts their navigation strategies under different navigation requirements, which is a great complement to the solution index of the dual-solution task.

To select the appropriate RL algorithms for the current study, we used temporal difference (TD) learning^9,11,12,18,21^, which is one of the most commonly used model-free algorithms in the RL literature. TD learning assumes that agents learn the future reward value following an action, and adjusts predictions continuously before obtaining the reward^5^. In the current study, we compared three types of TD models: TD(0), TD(λ) and TD(1). The major difference between TD(λ) and TD(0) is that TD(λ) adds an eligibility trace (see “Methods”), which adds the assumption that all the values of the visited locations are updated over time and the amount of updating depends on the visitation frequency. TD(1) is a special case of TD(λ) such that once a location is visited, the updated value at a location would never diminish over time even if it is not visited again. In other words, TD(1) assumes that there is no forgetting of the importance of the visited locations and therefore there is no need for a navigator whose cognition resembles this framework to revisit them to retain their relevance in wayfinding. We target these TD models because they differ from each other on how the importance of the visited locations changes over time—essentially, they make different assumptions about memory updating in spatial navigation. The topic of memory updating in spatial navigation has been studied extensively in experimental approaches^33,42–46^, but rarely through this computational lens. Note that these three TD algorithms predict that participants select different paths to reach destination based on their navigation history, but do not necessarily predict different navigation performance [e.g., one who favors TD(1) does not necessarily have to have better performance than the one who favors TD(0)].

In addition to model-free learning, we constructed a model-based model for spatial navigation. People who completely rely on a model-based system are assumed to have a perfect cognitive map^47,48^, and therefore one model-based model is sufficient. Because human performance relying purely on idealized cognitive maps may be implausible (at least for the vast majority of people) in many navigational scenarios, a hybrid model was constructed to reflect a heterogeneity or balance within an individual between map-like and more experience-bound knowledge. This hybrid model was developed by combing the best performing TD model with the model-based model, and adds a free weight parameter (ω) to capture the individual’s relative reliance on the model-based learning. As mentioned earlier, we hypothesized that the hybrid model was the best performing model in fitting human spatial navigation data.

The results from the model comparisons can inform us how well RL models fit the human spatial navigation literature, and as mentioned above, there are at least two parameters (navigation strategies and the consistency of using such strategies) generated from the RL models which could let us gain additional insight on human spatial navigation compared to traditional methods. To this end, we created navigation tasks with different requirements and investigated how these different navigational scenarios modulated navigation strategies and the consistency of using such strategies, which reflected how humans adapted to the ever-changing environment^12^ but has rarely been examined in the literature. Specifically, our design considers the fact that not every goal-directed navigation problem is best approached in the same way, and this creates a dynamic in which individual differences can also be understood in terms of how people shift their learning/behavioral model under different demands. In the current study, participants first found different objects in a virtual environment from a fixed starting location (the Fixed phase), and then found the same objects in the same environment but from various random locations (the Random phase). We were interested in how the relative model-based weight (ω) and the exploration–exploitation parameter (θ) changed as a function of learning experience and these different task requirements. In our hybrid model, ω represented the mean of an individual’s navigation strategy across a number of navigation trials, and θ represents the *consistency* of using this strategy in these trials (i.e., the degree to which an individual persists with a particular way of approaching the tasks in the face of feedback and changing demands).

Examining ω and θ separately and jointly would shed important light on how humans adapted to navigation scenarios of different requirements. Based on our manipulation of the navigation requirements, we hypothesized that (1) ω, the reliance on model-based system or the cognitive map, would increase from the Fixed to the Random phases due to increasing familiarity of the environment^19,22,23,49^ and the demands of the Random phase encouraging greater reliance on map-like knowledge. (2) Participants would be more exploratory or deviate from their default strategy more in the Random phase due to the randomness and uncertainty introduced^50^. Therefore, we hypothesized that θ would increase from the Fixed to the Random phases. (3) The correlations between ω and θ would be different in the Fixed phase from the Random phase—in the Fixed phase, where the starting location was always the same, there was no need to vary navigation strategy from trial to trial. In the Random phase, on the other hand, a more efficient strategy would be to rely on the model-free system when starting from a familiar location but to rely on the model-based system when starting from an unfamiliar location (thus favoring variation of navigation strategy). In other words, we theorized that better navigators would use one strategy more consistently in deterministic navigational scenarios, whereas they would vary their strategy more often in probabilistic navigational scenarios. From this theoretical perspective, we hypothesized that the correlation between ω and θ would be positive in the Fixed phase (i.e., better cognitive mappers would stick with one strategy more often than non-cognitive mappers), but the correlation would become negative in the Random phase (i.e., cognitive mappers may be more flexible in how they approach spatial problems). In this way, the hybrid RL model allows us to test a very specific but important prediction about the cognitive basis of human navigation performance. Finally, to show that ω was indeed reflective of spatial navigation ability, we correlated ω with objectively measured wayfinding performance, with the hypothesis that these two factors were significantly correlated.

To foreshadow our results, we found that the model-free model outperformed the model-based model in the Fixed phase, but vice versa in the Random phase. The hybrid model, on the other hand, was the best model of human navigation in both phases. Participants relied on cognitive maps more and deviated from their default strategy more in the Random than in the Fixed phases. Supporting our theoretical framework, the correlations between model-based reliance and exploration–exploitation were different between the Fixed and Random phases. Lastly, wayfinding performance was correlated with model-based reliance.

## Methods

### Participants

One hundred and twenty-six participants from Georgia Institute of Technology and the Atlanta community participated in this experiment, either for course credits or monetary compensation. Participants spent between 80 to 140 min completing the experiment. Twelve participants felt motion sensitive and did not finish the experiment. As a result, one hundred and fourteen participants (forty-six females) were included in the data analysis. A sensitivity power analysis showed that the smallest effect size our study could detect was r = 0.26 given our final sample size (114), targeting statistical power (0.8) and alpha level (0.05), which was sensitive enough to detect small (0.2) to medium (0.5) effects according to Cohen’s guidelines^51^. All participants (age 18–33) gave written consent and informed consent was obtained from all participants. The research was approved by the Institutional Review Board of Georgia Institute of Technology (IRB approval Code: H17456). All procedures were performed in accordance with the institutional guidelines.

### Materials and procedure

#### Navigation in virtual environment

Participants completed a practice session in a 4 × 4 grid of rooms to familiarize with the control scheme and the objective of the navigation task. The 3D virtual environment was created in Sketchup (www.sketchup.com) and the navigation task was rendered and implemented in Unity 3D video game engine (https://unity.com/). Each room was a square of 10 × 10 virtual meters in size with a wall of 3 virtual meter. There was a penetrable door in each side of the room except for the rooms at the boundary. Movement in the virtual environment was enabled by keyboard, which provided self-paced and continuous translation and rotation. After the practice session, participants started the Fixed phase.

##### Fixed phase

**Figure 1:**
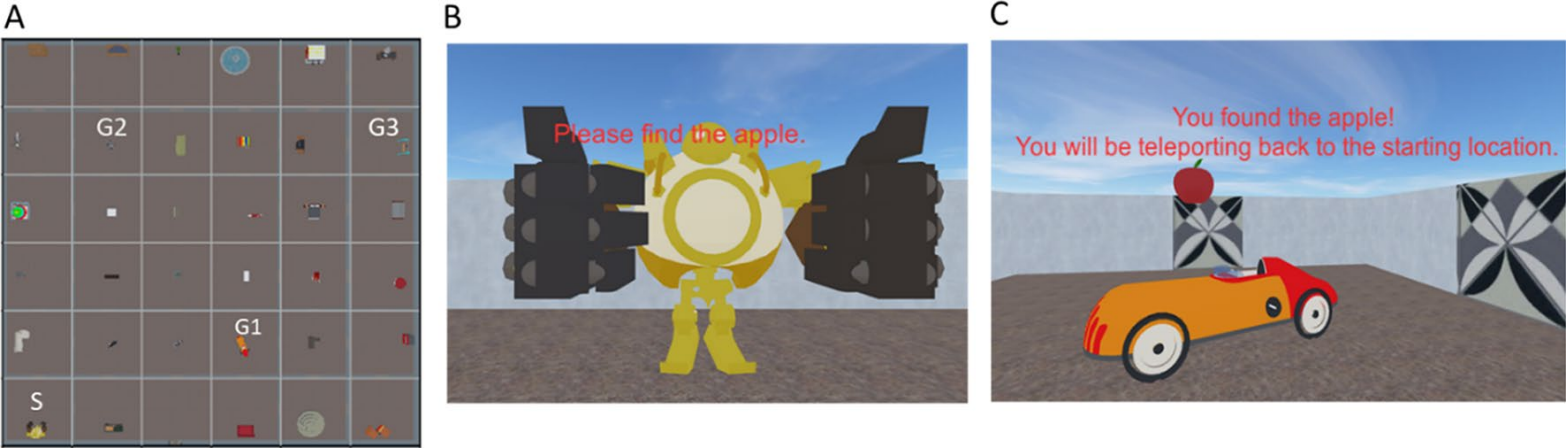
Experimental materials. (**A**) Layout of the environment. S indicates the fixed starting location in the Fixed phase, G1 ~ 3 indicate the goal object locations. (**B**, **C**) Actual view of landmark objects and rooms from the participants. Note that participants were brought back to the same starting location after finding a goal object during the Fixed phase.

To assess navigational learning and model it using RL algorithms, participants learned to navigate to hidden locations in a 6 × 6 grid of virtual rooms (Fig. 1). Each room had a unique reference object (toys, furniture, vehicles, etc.) served as local landmark which could only be seen within the room but not from other rooms. No distal or global landmark was available. Over the course of nine trials in the Fixed phase participants were instructed to find three specific goal objects repeatedly (apple, banana and watermelon; three trials per goal). These goal objects remained in the same rooms throughout the experiment but only the to-be-found goal object was invisible in a specific trial (e.g., all reference objects would be visible in their rooms, but if the goal object was “apple” in this trial, the banana would not appear even if participants traversed across the banana’s room). This helped avoid blending learning of different goal-destination pairings in the same trial. Once participants had found the goal object, they were teleported to the starting location and were instructed to find the next goal object. To make this Fixed phase amenable to model-free learning, participants were always brought back to the same starting position with the same facing direction, and each goal object was to be found in the same order across participants (i.e., apple-banana-watermelon and then repeat for all participants). This is akin to learning the outbound paths from one’s new home to the grocer, movie theater, etc. We limited the Fixed phase to nine trials to minimize the transfer of spatial learning, that is, many participants may start deriving shortcuts through model-based learning in the Fixed phase when they become extensively familiar with the environment^19,30,34^, potentially suppressing our ability to delineate interesting individual differences.

##### Random phase

Participants then underwent a “Random phase” in the same virtual environment as in the Fixed phase. Importantly, our implementation of a small number of trials in the Fixed phase in our design not only ensured participants did not derive and habitualize “shortcuts” during the Fixed phase but also still had room to improve their precise configural knowledge of the environment and continue learning at this point. Therefore, the Random phase represented a critical period involving a probe of (1) spatial transfer and flexible perspective adoption from the Fixed phase and then (2) continued environmental learning under new procedures. The Random phase was almost identical to the Fixed phase except that participants’ starting location and orientation were randomized in each trial (goal object locations were excluded in the possible starting locations). This is akin to finding the same grocer, movie theater, etc. from variable locations in the neighborhood. In addition, the order of goal objects was pseudorandomized such that each goal object was to be found once in every three trials but not in a predictable order (e.g., banana-apple-watermelon-apple-watermelon-banana…). There were seventy-two trials in the Random phase.

For our study, it was critical that the Fixed phase always preceded the Random for each participant. First, exposure to the Random phase prior to the Fixed phase may encourage participants to default to a model-based strategy and the performance in the Fixed phase could be at the floor level. Second, the Fixed phase—by repeating start-goal location pairings—enabled participants to develop one (or several) routes to a goal that would then be familiar in the Random phase and could be strategically exploited from a familiar landmark/room (enabling participants to exhibit shifts in strategy in Random where otherwise they would only have one [model-based] to go from).

### Analyses pipeline outline

Described in detail below, our analysis pipeline was as follows: we first fit each participants’ navigation behaviors with the three model-free models and selected the besting performing one (Fig. 3). We then created a hybrid model by combining the winning model-free model and a model-based model. Finally, we compared the performance of the model-free, model-based and hybrid models (Fig. 4) and chose the best performing model for subsequent individual differences analyses using its parameters (Table 1).

### Reinforcement learning models

**Figure 2:**
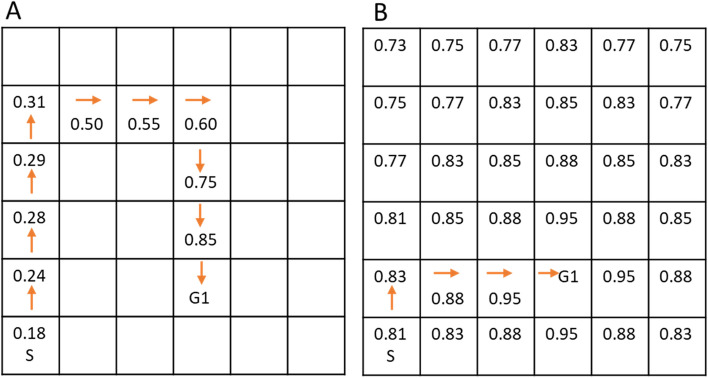
Model-free (**A**) and model-based (**B**) reinforcement learning models. The numbers in the figure are state values, showing how a navigator decides to move along the route in a given state/room. (**A**) Model-free valuation based on the TD algorithm. After finding an object this algorithm updates the values only along the traversed path. (**B**) Model-based valuations derived from dynamic programming. The model-based algorithm assumes a perfect cognitive map and the values in the entire environment are precomputed (See Model-based reinforcement learning). *S* starting location, *G1* Goal object # 1.

As mentioned in the Instruction, we used five different reinforcement learning models to fit navigation behaviors, separately for the Fixed and the Random phases and separately for each participant. We modelled the sequence of participants’ choices (which rooms to enter) by comparing them step by step to those predicted by various models. As we had a 6 × 6 grid, the navigation task consisted of 36 states (rooms) and in each state, subjects could have up to four action choices (up, down, left or right). The navigation task consisted of three rewards (three goal objects), and the objective for all models was to learn the state-action value function Q(s,a) at each state-action pair (i.e., which direction to go when in a specific room to maximize reward) for each goal object (Fig. 2). We assumed no interference or generalization among the (implicit) rewards of the three goal objects, and thus each algorithm was subdivided into three independent task sets and value functions, one for each goal object.

#### Model-free reinforcement learning

To provide further insight of model-free behaviors in human spatial navigation and chose the best one for the hybrid model, we created three TD models: TD(0), TD(λ) and TD(1). We first describe and provide the equations for TD(0), and then explain the differences between the three models. The equations for how the Q values were updated in the TD model were as follows^5^:

1 $${Q}_{TD}\left({s}_{t},{a}_{t}\right)= {Q}_{TD}\left({s}_{t},{a}_{t}\right)+ \alpha \delta $$

2 $$\mathrm{where }{\delta }= {r}_{t+1}+ {Q}_{TD}\left({s}_{t+1},{a}_{t+1}\right)-{Q}_{TD}\left({s}_{t},{a}_{t}\right)$$

Here, *t* denoted the current state and action, and *t* + 1 denoted the future state and action chosen by the softmax function (see below). Equation (1) showed that the Q value associated with the current state (Q(s_(t)_, a_(t)_)) was updated by an error δ, adjusted by the learning rate α. Equation (2) showed that the error δ was determined by the reward associated with the future state (r_(t+1)_) plus the difference between the Q values associated with the future and current states. The Q value of each state-action pair was initialized to be 0 in the beginning of the experiment, and the Q values were carried across trials and phases.

To determine which action to take based on Q values associated with the future states and actions, we computed the probability of the action selection based on the softmax function:

3 $${p}_{t+1}= \frac{{exp}^{\uptheta Q({s}_{t+1}, a)}}{{\sum }_{{a}^{^{\prime}}\in A}{exp}^{\uptheta Q({s}_{t+1}, a{^{\prime}}) }}$$

θ was the inverse temperature controlling the degree of randomness in participants’ action selection, and a′ denoted the possible future actions from the current state. θ was constrained between 1 to 15 based, and the higher the θ, the more deterministic of the action selection and therefore more exploitative.

Compared to TD(0), TD(λ) added the eligibility trace *e* to the Q value updating^5^, which was a temporary record of how frequently each state was visited. The eligibility trace for each state-action pair was set to be 0 in the beginning of each trial. The equations for how the Q values were updated in TD(λ) were as follows:

4 $${Q}_{TD(\lambda )}\left({s}_{t},{a}_{t}\right)= {Q}_{TD(\lambda )}\left({s}_{t},{a}_{t}\right)+ \alpha \delta {e}_{t}\left(\mathrm{s},a\right)$$

5 $$\mathrm{where } \, \, {e}_{t}\left(\mathrm{s},a\right)=\lambda {e}_{t-1}\left(\mathrm{s},a\right)+\mathbf{I}({S}_{t}=s, {A}_{t}= a)$$

**I** was the indicator function, which was equal to 1 when the condition inside it was true and 0 otherwise. λ was constrained between 0 and 1, so Eq. (5) indicated that the less frequently a state was visited, the smaller the updating of the Q value associated with that state. TD(1) was a special case of TD(λ), which forced every visited state to get the same amount of updating regardless of how often they were visited. When relating these RL algorithms to human memory systems, TD(0) assumed that memory updating, which was represented in the Q value updating, occurred only in the most recent visited location, whereas TD(λ) assumed that memory updating occurred in all previously visited locations continuously and such updating scaled with the frequency of visitation. TD(1) differed from TD(λ) that memory updating did not scale with the frequency of visitation.

#### Model-based reinforcement learning

For the model-based algorithm, we used dynamic programing^5^ which learned the layout of the environment (i.e., cognitive map) by computing the Q values via traversing all possible rooms and directions to locate the goal (Fig. 2). We computed the Q values based on a ‘sweeping’ process terminating at goal locations. We first initialized all Q_MB_(s) to 0 in the beginning of the Fixed phase. Then, for all states and adjacent state-action pairs we iteratively performed the following:

6 $${Q}_{MB}(s)\leftarrow {\sum }_{a}\pi (a|s)+{\sum }_{{s}{^{\prime}},r}p\left({s}{^{\prime}},r|s,a\right)\left[r+ \gamma {Q}_{MB}\left({s}{^{\prime}}\right)\right]$$

where $(a|s)$ was the probability to take action *a* from state *s* following the exploration vs. exploitation policy. $p\left({s}^{^{\prime}},r|s,a\right)$ was the probability to end up in state *s*′ and receive reward *r* given the current state and action. The algorithm had one fixed parameter γ set at 0.8. The final model-based values (Q_MB_) were the values after the algorithm converged (i.e., the difference between each of the Q_MB_ in the current iteration and the previous iteration was smaller than 0.0001). Conceptually, the model-based values reflected the state-action values as if one had a perfect cognitive map, and therefore the Q values did not get updated and they were the same for all participants.

#### Hybrid model

We implemented a hybrid model as a weighted linear combination of the values from the best performing model-free algorithm across participants and the model-based algorithm:

7 $${Q}_{hybrid}=\left(1 -\upomega \right){Q}_{MF}+ \omega {Q}_{MB}$$

where ω represented the balance between the model-free and model-based behaviors. The higher the ω, the better the navigation behaviors could be characterized as model-based or cognitive map guided.

#### Model fitting and comparison

For each algorithm, we computed the negative log-likelihood (NLL) of the observed choices (${a}_{t}$) by the summing over the log of Eq. (3), for the action chosen on each of the n trials, as follows:

8 $$NLL\left({\varvec{X}}\right)=-\sum_{t=1}^{n}logp\left({a}_{t}|{\varvec{X}}\right)$$

where vector **X** denotes the free parameters of the model, and the NLL was computed separately in Fixed and Random phases. The best fitting parameters were then computed as those that minimize the negative log likelihood:

9 $${{\varvec{X}}}_{\mathrm{MLE}} = \mathit{arg} \,\underset{x} {\mathrm{min}} \, NLL({\varvec{X}})$$

Model fitting was performed using the the optimization function from SciPy^52^. Model comparison was performed by computing the Bayes Information Criterion (BIC) for each model for each participant, separately in the Fixed and Random phases.

10 $$BIC = \mathit{klogn}+2{{\varvec{X}}}_{\mathrm{MLE}}$$

where k is the number of free parameters in the model and n is the number of trials in the data. There were two free parameters (α and θ) in the TD(0) and TD(1) models, and three free parameters (α, θ and λ) in TD(λ). There was one free parameter θ in the model-based model, and four free parameters (α, θ, λ and ω) in the hybrid model.

### Excessive distance

Excessive distance (ED) has been a widely used index to indicate wayfinding efficiency^19,22,49,53^ which was defined as:

$$({\text{actual}} \, {\text{traversed}} \, {\text{distance}} - {\text{optimal}} \, {\text{distance}}) / {\text{optimal}} \, {\text{distance}}.$$

An ED of 0 indicated perfect wayfinding performance (actual traversed distance equals to optimal distance) and an index of 1 indicated the actual traversed distance was 100% longer than the optimal distance. In our study, because states and state transitions were compartmentalized by rooms, we used the number of rooms to represent distance.

## Results

We used JASP (JASP Team, 2021) and Cocor^54^ for statistical analyses, and Matplotlib^55^ and Seaborn for data visualization.

### TD(λ) outperformed other TD models in fitting spatial navigation behavior

**Figure 3:**
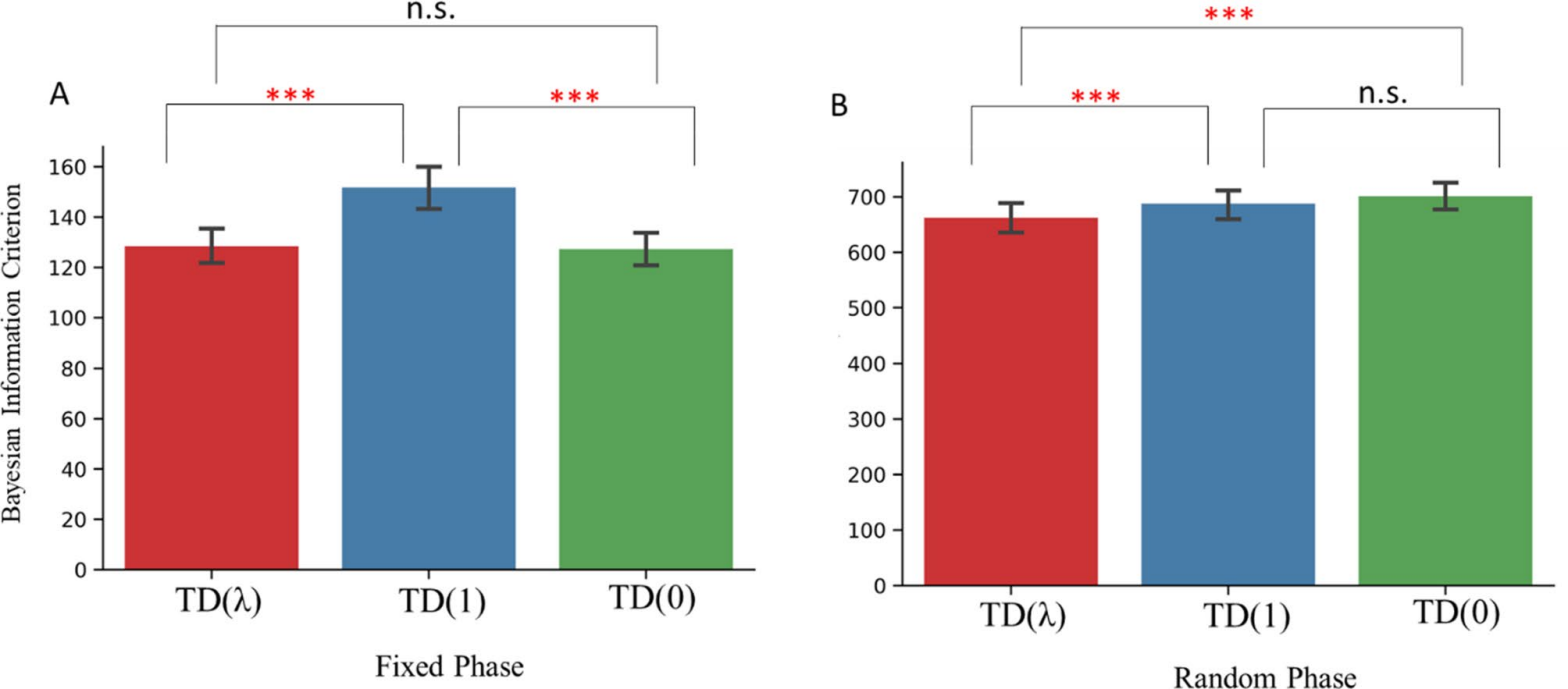
Model comparison in the Fixed (**A**) and the Random (**B**) phases. *BIC* Bayesian Information Criterion. *n.s.* not significant. ***p < 0.001.

We first compared the TD family algorithms in modeling navigation behavior in the Fixed and Random phases, separately (Fig. 3). In the Fixed phase, the one-way repeated ANOVA, with the three TD models as independent variable and BIC as dependent variable, was significant (*F*(2,226) = 49.77, *p* < 0.001, η^2^ = 0.30). Paired t-test showed that TD(λ) outperformed TD(1) (*t*(113) = − 7.04, *p* < 0.001, Cohen’s *d* = − 0.66), and was similar to the TD(0) model (*t*(113) = 0.94, *p* = 0.35, Cohen’s *d* = 0.09). The TD(0) model outperformed the TD(1) model (*t*(113) = − 7.59, *p* < 0.001, Cohen’s *d* = − 0.711; Fig. 3A). In the Random phase, the one-way repeated ANOVA was also significant (*F*(2,226) = 10.48, *p* < 0.001, η^2^ = 0.09). Paired t-test showed that TD(λ) outperformed TD(1) (*t*(113) = − 3.62, *p* < 0.001, Cohen’s *d* = − 0.34), and the TD(0) model (*t*(113) = − 4.52, *p* < 0.001, Cohen’s *d* = − 0.42). There was no significant difference between the TD(0) and the TD(1) models (*t*(113) = 1.36, *p* = 0.18, Cohen’s *d* = 0.13; Fig. 3B). Overall, the TD(λ) was the best performing model among our selection of model-free models, and therefore we used it for the hybrid model.

### The hybrid model outperformed the model-free and model-based models in both phases

**Figure 4:**
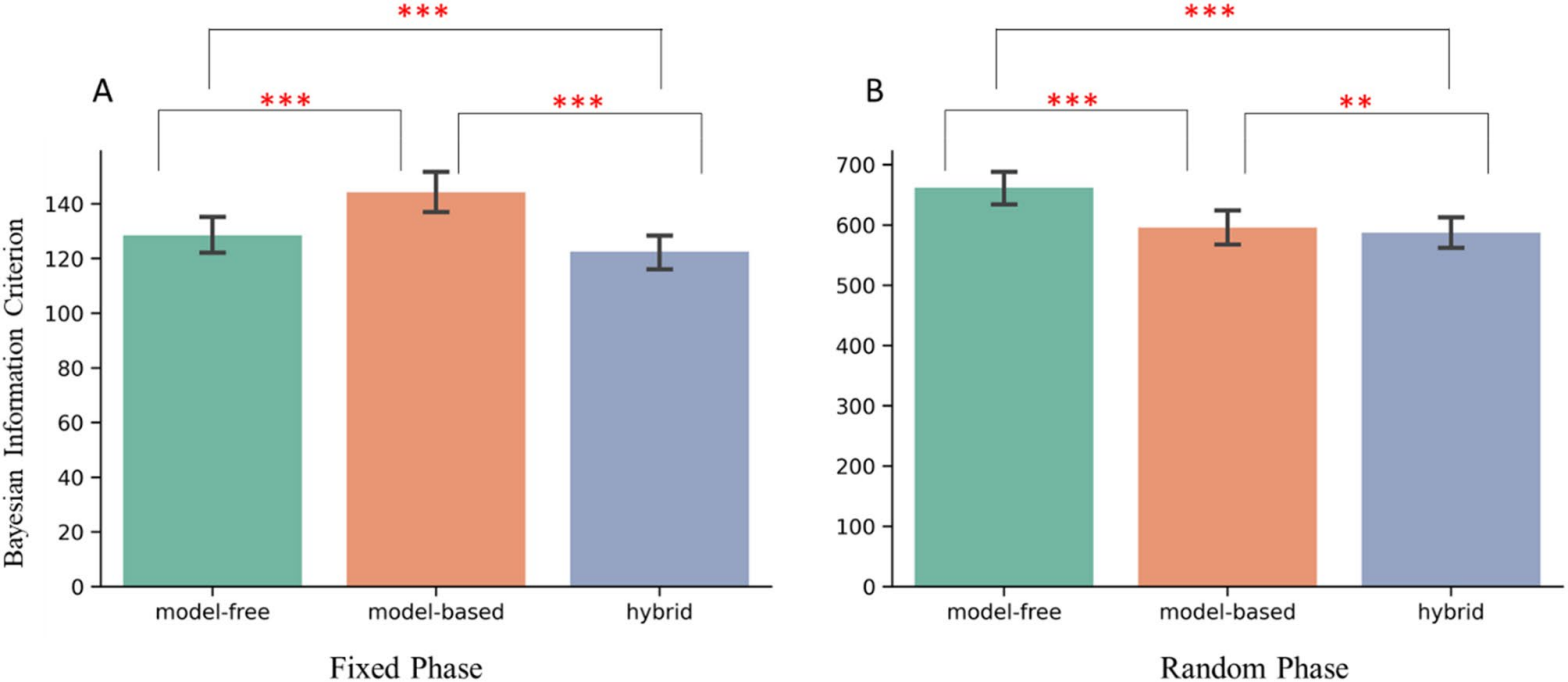
Model comparison in the Fixed (**A**) and the Random (**B**) phases. *BIC* Bayesian Information Criterion. **p < 0.01, ***p < 0.001.

We next compared which model, namely the model-free, model-based and the hybrid, provided the best fit in the Fixed and Random phases, respectively (Fig. 4). In the Fixed phase, the one-way repeated ANOVA was significant (*F*(2,226) = 105.09, *p* < 0.001, η^2^ = 0.48). Paired t-test showed that the hybrid model outperformed the model-free (*t*(113) = − 4.07, *p* < 0.001, Cohen’s *d* = − 0.38), and the model-based models (*t*(113) = − 17.93, *p* < 0.001, Cohen’s *d* = − 1.68). Furthermore, the model-free model outperformed the model-based model (*t*(113) = − 8.34, *p* < 0.001, Cohen’s *d* = − 0.78; Fig. 4A). In the Random phase, the one-way repeated ANOVA was also significant (*F*(2,226) = 114.74, *p* < 0.001, η^2^ = 0.50). Paired t-test showed that the hybrid model outperformed the model-free (*t*(113) = − 11.62, *p* < 0.001, Cohen’s *d* = − 1.09), and the model-based models (*t*(113) = − 3.07, *p* = 0.003, Cohen’s *d* = − 0.29). Contrary to the results in the Fixed phase, the model-based model outperformed the model-free model in the Random phase (*t*(113) = − 10.59, *p* < 0.001, Cohen’s *d* = − 0.99; Fig. 4B). Clearly, the hybrid model the best performing model in both navigational phases.

**Table 1:** Correlation matrix between navigation strategy (ω) and exploration–exploitation (θ) in the Fixed and Random phases.

	1	2	3	4
1. Fixed ω	–			
2. Random ω	0.53***	–		
3. Fixed θ	0.25**	0.31***	–	
4. Random θ	− 0.27**	− 0.35***	− 0.20*	–

*p < 0.05, **p < 0.01, ***p < 0.001.

### The correlation between navigation strategy (ω) and exploration–exploitation tendency (θ) was modulated by navigation requirement

Lastly, we asked whether navigation strategy (ω) and exploration–exploitation (θ) was modulated by navigation requirements. ω was significantly smaller in the Fixed than in the Random phases (*t*(113) =  − 17.56, *p* < 0.001, Cohen’s *d* = − 1.64), suggesting that in general, participants’ navigation behaviors reflected more model-free in the Fixed than in the Random phases. On the other hand, θ was significantly larger in the Fixed than in the Random phases (*t*(113) = − 7.75, *p* < 0.001, Cohen’s *d* = 0.73), suggesting that in general, participants used the same navigation strategy more consistently in the Fixed than in the Random phases. We then compared the correlations ω and θ in the Fixed and Random phases (Table 1). In the Fixed phase, this correlation was significantly positive (*r*(114) = 0.25, *p* = 0.007), suggesting that in the Fixed phase, the cognitive mappers tended to exploit or used the same navigation strategy more consistently than the route followers. In the Random phase, on the other hand, this correlation became significantly negative (*r*(114) = − 0.35, *p* < 0.001), suggesting that in the Random phase, the cognitive mappers tended to explore or vary their navigation strategy more than the route followers. Together, these results supported our theoretical framework of one important way that cognitive mappers differ from route followers: cognitive mappers are flexible and efficient not only by virtue of making use of cognitive map-based strategies, but by adaptively avoiding or embracing strategy change based on different navigational requirements.

To demonstrate that ω was also correlated with objectively observed performance, we correlated ω and excessive distance (ED). We found that ω was correlated with ED significantly in both Fixed and Random phases (*r*s(114) < − 0.51, *p*s < 0.001), supporting the prediction that regardless of navigational requirements, more model-based behavior is indicative of being a better, more spatially-efficient navigator.

## Discussion

The current study compared five RL models in characterizing human behaviors in navigation tasks with different requirements, and we found that a hybrid model, consisting of both model-free and model-based learning, provided the best fit in both navigation tasks, despite being penalized (in model comparison) for its greater complexity. Furthermore, through individual differences analyses, we found that the reliance on the model-based system (ω) and the variability of using the default strategy (θ) increased as the randomness of the wayfinding increased. Interestingly, the correlation between ω and θ was modulated by task requirements, such that individuals who relied more on model-based learning were more likely to stick with one navigation strategy when wayfinding was from the same starting location, but were more likely to vary their navigation strategy when wayfinding was from an unpredictable starting location.

We first compare three model-free models, namely the TD(0), TD(λ) and TD(1), to determine the role of memory updating in spatial navigation. As mentioned in the Introduction and Methods, TD(0) assumes memory updating only occurs in the most recent visited location. On the other hand, the eligibility trace in TD(λ) assumes that memory updating occurs in all previously visited locations and the amount of updating decreased over time if such locations were not visited again. TD(1) is the special case of TD(λ) that memory updating is the same in all previously visited locations regardless of their visitation frequency. Our results show that although the TD(λ) model is not better than the TD(0) model in the Fixed phase, it does outperform the other model-free models of our navigators’ cognition in the Random phase. As evidenced by the superiority of the hybrid model in this phase over a purely model-based approach, and by virtue of TD(λ)’s properties, these findings suggest that to the extent that people exhibit TD-like profiles their spatial memory updating typically occurs in a more continuous manner across all previously visited locations and scales with visitation frequency in spatial navigation, especially when wayfinding is not completely deterministic (i.e., the Random phase). Our findings not only complement the literature on memory updating in spatial navigation^33,42–46^, but also extend these findings via a computational approach.

As stated in the Introduction, the increasing familiarity of the environment and the demands of the Random phase would encourage participants to rely on map-like knowledge in a greater extent in the Random phase. Indeed, when compared the performance of model-free learning against model-based learning, we find that the model-free learning outperforms model-based in the Fixed phase, but is outperformed by model-based in the Random phase, which validates our modeling methods. The hybrid model, on the other hand, outperforms the model-free and model-based models in both learning phases, suggesting that the majority of the individuals did not entirely rely on either the model-free or model-based learning systems, in either scenario, but instead fell somewhere in between. These findings are aligned with the well-established findings of the substantial individual differences in spatial navigation, such that—although some individuals have little or near perfect configural knowledge of their environment—most of fall somewhere in between on various objective measures^22–24,26,36,56^. To the best of our knowledge, this is the first study showing that a hybrid RL model significantly outperforms the model-free and model-based models in human spatial navigation explicitly.

After confirming that the results from the hybrid model were in line with the findings in the literature, we extracted the two key parameters to give a new understanding of individual differences in spatial navigation in such tasks. A large portion of the literature in human spatial navigation investigates what makes a good navigator^57^, and a critical component is navigation strategy (route-following or cognitive mapping)^29–31,34^. As mentioned in the “Introduction”, the most widely used method of measuring one’s navigation strategy is the dual-solution task, which may simplify the complex navigation behaviors observed in humans. The parameter ω of the hybrid model indicates the proportion which people use a cognitive map in spatial navigation relative to following familiar routes, and it is significantly correlated with, but importantly is not identical to, navigation performance. The critical difference between ω and the solution index from the dual-solution task is that the solution index indicates a general navigation strategy as a trait over a number of trials, but ω can be used to indicate a general strategy (like what we did in the current study), but also be used in a trial-by-trial basis where each trial has its own ω. In other words, the RL models can provide a finer grained measure of one’s navigation strategy compared to traditional methods.

Another important and unique contribution of the RL models is the parameter of the exploration–exploitation tendency (θ), which reflects how consistently one uses their default strategy and is very difficult to estimate with traditional methods. The combination of ω and θ provides a powerful and unique way to further understand what contribute to the substantial individual differences in spatial navigation. For example, in our study the change in correlation between ω and θ reveals that cognitive mappers use different strategies more often when the randomness in the task increases, which is a novel finding on what makes a good navigator^57^. This finding is also important because although model-based behavior itself captures navigational flexibility, we also see that people with good cognitive maps and an ability to engage in model-based behavior are also the same people who more often appropriately shift between frameworks For example, imagine rounding a corner and a familiar set of cues becomes visible—it may be that a familiar path forward from this point, aligning with model-free behavior, is in fact the most efficient option, and our computational approach reveals how a good cognitive mapper may make this switch. Taken together, these findings not only give us a new perspective for understanding the individual differences in human spatial navigation ability, through the lens of navigation strategies and the consistency of using such strategies, but also have important implications for how one might improve spatial navigation ability in humans as well as artificial agents.

## Limitations

In the current study, the Fixed phase always preceded the Random phase. As elaborated in the “Methods”, this was particularly important for the design and current research questions—however, an obvious step for subsequent studies would be to leverage a longer design that includes switches back to Fixed trials (either interleaved with Random or blocked) at different stages of learning, in order to understand how the experience of navigating our environment in more flexible ways (Random) may influence our internal model used when navigating more familiar relationships between locations.

Another important design consideration was that the environment layout was a regular grid-shape without any global landmarks. These attributes combine to make orientation in the global space more difficult and dependent on learning relationships between adjacent rooms. On the one hand, we view this as a strength of the design, especially in the context of studying state-to-state associations in a reinforcement learning model. And indeed, there are many real-world scenarios that share features with our virtual environment—navigating enclosed spaces, such as the interior of a hotel, mall, or hospital, and to a lesser extent subway maps. In addition, when nestled down among tall buildings in city streets our view of landmarks are more likely to be constrained to vista space and a local scale^58^, somewhat akin to the present task’s constraints (but not wholly). Nonetheless, another obvious step forward from this research is to investigate whether the shape of the environment and the presence of global landmarks affects the pattern of results reported in the current study. One might hypothesize that—by facilitating shortcutting—global landmarks would reduce the propensity of some people to exploit familiar route segments in lieu of exploration at the Random phase transition. On the other hand, pivoting from some of our other recent findings^22^, it may also be the case that better navigators are those who flexibly take advantage of these additional cues more-so than poorer navigators, in which case an intriguing prediction from the present findings is that such cues may exacerbate differences between more and less-model-based individuals, and their variability in strategy, according to both task demands and cue availability. Future studies could test these complementary ideas.

## Conclusions

In the current study we compare five reinforcement learning models in fitting human spatial navigation behaviors. We find that the hybrid model provides the best fit to the data regardless of task requirements, and it sheds important light on how task requirement modulates the navigation strategy (the balance between model-free and model-based), the consistency of using, and the interaction between these two factors. All in all, we show that reinforcement learning models provide a finer grained characterization of navigation strategy in a continuous manner based on individual’s prior navigation history, which not only complements and extends the existing methods of studying individual differences in spatial navigation, but also suggests that the consistency of using a navigation strategy based on navigation requirements is an important factor of what makes a good navigator.

## Data Availability

The data that support the findings of this study and the analysis code are available from the corresponding authors upon request.
